# Cannabis dependence: Effects of cannabis consumption on inter-regional cerebral metabolic relationships in an Indian population

**DOI:** 10.4103/0019-5545.70976

**Published:** 2010

**Authors:** Shubhangi R. Parkar, Seethalakshmi Ramanathan, Narendra Nair, Shefali A. Batra, Shilpa A. Adarkar, Anirudh G. Pandit, Purushottam Kund, Nawab Singh Baghel

**Affiliations:** Department of Psychiatry, Seth GS Medical College and King Edward Memorial Hospital, Mumbai, India; 1 Radiation Medicine Centre, BARC, Mumbai, India

**Keywords:** Cannabis, 2-fluoro, 2-deoxy-glucose positron emission tomography, inter-regional metabolic relationships

## Abstract

**Background:**

The effects of cannabis consumption on neurophysiological function have been a matter of considerable debate. With the legalization of medical marijuana, understanding the consequences of cannabis dependence has become extremely important.

**Aim:**

We attempted to understand the influence of cannabis on cerebral glucose metabolism in certain predetermined regions of interest (ROIs). Furthermore, we also explored inter-regional metabolic relationships between ROIs forming the “addiction” and “cognitive dysmetria” circuit.

**Materials and Methods:**

2-fluoro, 2-deoxy-glucose positron emission tomography (FDG PET) scans were carried out in 16 male patients (age: 25.3±10.38 years) with cannabis dependence, 8–12 hours after the last cannabis consumption. Resting glucose uptake in 14 pre-determined ROIs was compared with glucose uptake in 16 non-drug using volunteers (age: 29.2±8.39 years).

**Results:**

The two groups differed in their lateral and medial temporal glucose uptakes by approximately 16–24%. The relationships between inter-regional glucose uptakes in the two circuits were compared using the Chow Test. Significant differences in inter-regional correlations in the medial temporo–frontal and parieto–thalamic were noted between the two groups.

**Conclusion:**

The altered metabolic relationships among various brain regions can have potentially important implications for understanding cannabis dependence and cannabis-induced psychopathology.

## INTRODUCTION

The effect of cannabis on neurophysiological functioning has been a topic of interest for many years. Recent evidence of possible schizophrenogenic potential has brought cannabis back into the limelight. On the other hand, medical marijuana is also gaining rapid interest for its “neuroprotective” properties. It has, therefore, become essential to affirm the neuropsychiatric effects of cannabis. Furthermore, in some developing societies such as India, the use of cannabis enjoys religious sanction. Indeed, India has seen a significant rise in the prevalence of cannabis dependence. A national survey, carried out in 2003, noted that approximately 8.75 million people reported using cannabis. Of these, only a quarter (or 2.3 million) had ever sought treatment for cannabis use.[1] Considering the magnitude of cannabis dependence and possible serious neuropsychiatric consequences, a neuroimaging study examining the influence of cannabis on cerebral glucose metabolism in an Indian population is clearly warranted; however, none has been carried out till date.

### Effects of cannabis on glucose metabolism

Cannabis acts through CB1 receptors that have been identified in varied regions of the brain, including the cerebellum, basal ganglia and the hippocampus.[2] A number of functional imaging studies have identified a wide range of changes in cerebral functioning in cannabis users in various stages of cannabis use and dependence.[3,4] Functional imaging studies examining individuals with cannabis dependence during periods of withdrawal are few. Block *et al*.[5] examined cerebral metabolic rate in cannabis users following 26 hours of abstinence, a period that can be considered as subclinical withdrawal. They noticed that on a memory-based task, individuals with cannabis dependence demonstrate different activation patterns as compared to non-using controls. A second functional magnetic resonance imaging (fMRI) study[6] conducted within 6–26 hours of last cannabis consumption noted that individuals with cannabis dependence recruit more areas on a spatial working memory task as compared to normal non-drug using controls. Based on these, we formed our first hypothesis.

#### Hypothesis 1

Individuals with cannabis dependence have different regional glucose metabolisms as compared to non-drug using controls. As we planned to avoid the immediate effects of cannabis intoxication, we selected a time frame of 8–10 hours after last cannabis consumption.

### Influence of cannabis on neural networks

Cannabis has been identified as having “seemingly contradictory neurotoxic and neuroprotective effects”.[7] It has been suggested that cannabis can act as a modulator of synapses and, thus, potentially disrupts neural networks. Most studies examining neural networks have primarily used blood oxygenation-level dependent-fMRI (BOLD-fMRI) measurements. For our 2-fluoro, 2-deoxy-glucose positron emission tomography (FDG PET) measurements, we assumed that regions that are part of a circuit/network would share a specific and directional metabolic relationship that enables adequate functioning of the circuit. We explored regions involved in two circuits. The first one is the circuit involving the frontal, medial temporal and thalamus that has been implicated in substance dependence.[8] Further, considering the controversial role of cannabis in schizophrenia, we investigated the circuit for cognitive dysmetria in schizophrenia – cortical–subcortical–cerebellar circuitry.[9] This forms the basis of our next three hypotheses.

#### Hypothesis 2

There would be significant differences in inter-regional metabolic patterns between individuals with cannabis dependence and non-drug using volunteers in the substance dependence circuit.

#### Hypothesis 3

As neither individuals with cannabis dependence nor non-drug using volunteers have any symptoms suggestive of schizophrenia, there should be no differences in the cortical–subcortical–cerebellar circuitry.

## MATERIALS AND METHODS

The study was conducted by the de-addiction center (psychiatry department) of a tertiary care hospital in collaboration with the Radiation Medicine Centre, Bhabha Atomic Research Centre, during January 2004 – December 2005. Approval for the study was obtained from the Institutional Review Boards of both the institutions.

### Subjects

Sixteen male patients with a DSM-IV diagnosis of cannabis dependence[10] and ongoing cannabis consumption were invited to participate in the study (Group I). The diagnosis was made by two qualified psychiatrists using a semi-structured interview of the participant and corroborated with a reliable family member/guardian. Comorbid Axis I diagnosis, benzodiazepine intake in the last 6 months, past or current history of any neurological or medical illness were ruled out. The control group (Group II) comprised 16 consenting right-handed, non-drug using male volunteers with no history of past or present Axis I diagnosis. Only males were invited to participate in the study to avoid all possible gender confound.

Consenting participants completed a semi-structured questionnaire detailing duration and amount of cannabis consumption. Cannabis consumption was confirmed by urine thin layer chromatography, prior to participation in the study. No quantitative assessment was, however, carried out. All the participants smoked cannabis in cigarettes. None of the participants in either group had any past or present history of other substance dependence, except nicotine. Exclusion of other substance dependence, including opiates and alcohol, was confirmed by urine drug screening. Further, as the participants were not in a controlled environment prior to the scan, these urine toxicology screens were repeated prior to the scan.

Study participants continued their ongoing pattern of consumption, their last consumption varying from 10 to 12 hours prior to the scan. At the time of the scan, none of the participants reported any symptoms of cannabis intoxication or withdrawal. Subjects were also requested to refrain from nicotine and caffeine for at least 8 hours prior to the scan.

### FDG PET procedure

The standard FDG PET protocol was followed. F18-FDG[11] is produced in a 16.5-MeV Medical Cyclotron Facility located in the center that hosts the scanner (Radiation Medicine Center, BARC, Mumbai). Participants were fasting for at least 8 hours prior to the scan and showed a blood glucose level of <150 mg/dl. An average dose of 200 MBq (160–230) of F18-FDG was injected. Acquisition was carried out 30 minutes after the injection. Positioning was achieved with the help of LASER align lights and head was secured with restraints to minimize artifacts due to movement. The pattern of cerebral glucose metabolism was examined using F-18-FDG with a GE Advance PET System scanner NXI (General Electric Medical Systems, Milwaukee, WI, USA). The scanner has a transaxial resolution of 4.8–6.2 mm full width half maximum (FWHM) depending upon the distance from the center and an axial resolution of 4.0–6.6 mm FWHM. Emission scans of 70 slices were obtained parallel to the cantho-meatal line from vertex to the neck. Transmission scans were obtained for the same region using Ge-68 rod sources to carry out measured attenuation correction. The images were reconstructed using the Ordered Subsets Extraction Maximization (OSEM) algorithm. These images were reformatted and converted into 35 transaxial slices of 4.25 mm thickness.

### Analyses

Regional glucose metabolism was examined in 14 pre-determined Regions of Interest (ROIs) – elliptical ROIs for cortical and subcortical structures and circular ROIs for cerebellar hemispheres. For the purpose of selection of ROIs, the slice of the brain through the basal ganglia was taken as reference slice. One slice above and below was checked for maximum uptake values (mUVs) for each ROIs. For the cerebellum, midcerebellar slice was selected. ROIs considered were the following:

- prefrontal regions (right and left),
- temporal (right, left, medial and lateral),
- parietal (right and left),
- occipital (right and left),
- basal ganglia (right and left),
- thalamus (right and left) and
- cerebellum (right and left).

The regional activity in a given ROI was measured as the mUVs in that ROI (kBq/ml).

**Figure 1:**
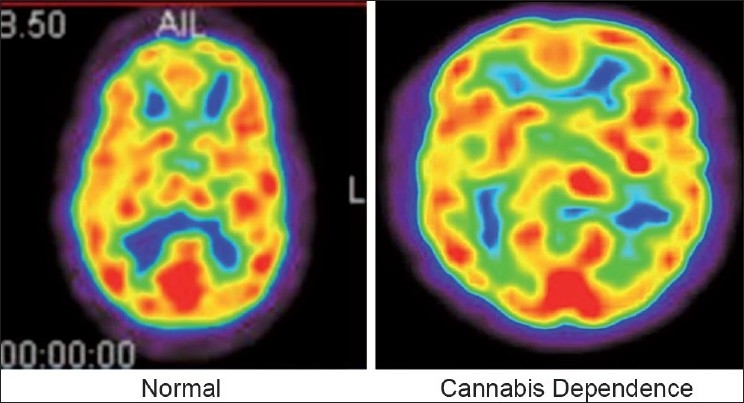
Representative PET images from single subjects of each group illustrating differences in regional glucose uptakes among the various brain regions

During qualitative assessment, it was observed that in a majority of the patients, the occipital lobes showed maximum FDG uptake. Additionally, most studies examining influence of cannabis on Cerebral blood flow have failed to report any changes in the occipital lobe in cannabis users [Table 1]. Figure 1 provides illustrative images from each group demonstrating the uptakes in the various brain regions. These are not SPM images. These are normalized images, however from single participants of each group. Hence, the average of the occipital lobe activity uptake values was taken as the normalizing factor. Hence, results related to these regions were not reported. The glucose uptakes in the other ROIs were expressed as relative uptake values (rUVs) – ratio of uptake value for ROI to average uptake value for the occipital lobes. Analysis was carried out using these rUVs in the various ROIs.

**Table 1:** Relative glucose metabolism (rUV) in cannabis users compared to non-drug using controls: Mean values depicted (independent sample *t*-test) (df – 30)

ROI	Cannabis dependents	Non-drug using controls	*t*-value	*P*-value
R frontal	76.00	85.78	−2.19	0.51
L frontal	76.60	84.70	−1.93	0.88
R parietal	78.51	85.90	−1.89	0.95
L parietal	76.13	83.30	−2.09	0.64
RL temporal	79.90	67.70	3.06	0.064
LL temporal	77.63	62.56	4.17	0.003*
RM temporal	61.03	75.34	−4.1	0.003*
LM temporal	61.14	73.18	−3.87	0.007
R cerebellum	71.41	75.93	−1.12	1
L cerebellum	69.86	80.37	−2.35	0.35
R basal ganglia	81.66	88.69	−1.43	1
L basal ganglia	84.44	88.36	−0.71	1
R thalamus	83.58	86.53	−0.57	1
L thalamus	82.44	83.92	−0.33	1

* Significant at the 0.05 level (2- tailed); with Bonferroni correction (*P* 0.05/14-0.004)

Data were analyzed using STATA/SE 9.2 (Stata Corporation, College Station, TX, USA).

Finally, in order to understand differences in the inter-regional correlations of the rUVs, we used linear regressions combined with Chow Tests to test if the correlation between two regions were different between cannabis users and non-drug using controls. As an example, when examining the efferent relationship from frontal to medial temporal ROIs, we fitted two linear regressions, one for each group, with frontal rUVs as the independent variable and medial temporal rUVs as the dependent variable with an age correction (the age correction was used to avoid any confounding influences of age on inter-regional metabolic relationships). The Chow Test is a Chi-square test used to test whether the slope coefficients in these two regressions are statistically different. In the above example then, a positive test indicates that the efferent relationship from the frontal ROI to the medial temporal ROI is statistically different between the two groups.

## RESULTS

### Sample characteristics

The mean age in both groups [Group I: 25.25 years (SD: 10.38, range: 16–50); Group II: 29.55 years (SD: 8.39, range: 18–48)] was comparable (*P*>0.05). The mean age at onset of cannabis consumption was 16.2 years (SD: 3.6 years, range: 10–23 years); duration of consumption varied from 6 months to 40 years (mean: 8.6 years). All participants smoked cannabis in cigarettes. The patients spent an average of Rs. 60.60 per day (Rs. 20–Rs. 150) (1 British pound was approximately Rs 80 during the period of the study). This amount spent on cannabis is reflective of the quantity of cannabis consumed, in terms of potency. We have chosen not to involve the amount of cannabis consumed in weight as this would not have been a true reflection of the potency of cannabis. In India, the rates of cannabis (at the time of this study) ranged from Rs. 10 to Rs. 50 for 10 g depending on the quality of cannabis. In other words, a user might be consuming smaller quantities of unadulterated, yet pure, cannabis with higher potency. Daily consumption in our sample ranged from 10 to 30 g of cannabis.

### Regional glucose metabolism in cannabis users compared to non-drug using controls (independent sample *t*-test with Bonferroni correction)

Mean rUVs were higher in bilateral lateral temporal regions in the cannabis dependent group as compared to non-drug using controls. The higher metabolism in the left lateral temporal region remained statistically significant with the Bonferroni correction (*P* 0.003). In all the other ROIs examined, rUVs were lower in the cannabis dependent group than in the non-drug using controls. After correcting for multiple comparisons, this decrease was statistically significant in the medial temporal regions (*P* 0.003, 0.007). We further ascertained that these differences were not influenced by age, using regression analysis [Table 1] [Figure 1].

### Influence of duration and amount of cannabis consumption on glucose uptake (Group I) (Pearson’s correlation with Bonferroni correction)

Significant correlations were noted between duration of cannabis consumption and right cerebellar uptake; amount of cannabis consumption correlated significantly with right parietal and occipital glucose uptake values. However, none of these significances survived Bonferroni correction for multiple comparisons. An additional correlation was attempted with a composite cannabis consumption (CCC) value (a measure of estimated lifetime cannabis consumption). This was derived as the product of amount of cannabis consumption with duration of cannabis consumption. No significant correlations were noted between this CCC and glucose uptake in any of the ROIs. Age at first consumption did not influence glucose uptake in any ROI.

### Inter-group differences in metabolic relationships among the different ROIs

#### Metabolic relationship between the frontal–medial temporal–thalamus ROIs implicated in addiction

When we examined the relationships between regions implicated in addiction (frontal–medial temporal and frontal–thalamus), the afferent and efferent relationships between frontal, medial temporal regions and the thalamus were significantly different. However, after correcting for multiple comparisons (Bonferroni correction), only the relationship between the frontal and the medial temporal regions (*P* 0.05, 0.02) remained significant, albeit with a hemispheric difference Table 2.

**Table 2:** Differences in inter-regional metabolic relationships in the motive circuit for substance dependence (Chow test with Bonferroni corrections)

Afferent	Efferent	Regression slope coefficients		*P*-value
		Cannabis	Non-drug using controls	
Frontal	Thalamus			
Right		0.29	0.82	0.21
Left		0.11	0.61	0.12
Thalamus	Frontal			
Right		0.20	0.37	0.94
Left		0.26	0.82	1.00
Frontal	M Temporal			
Right		0.46	1.09	0.56
Left		0.45	1.31	0.02*
M Temporal	Frontal			
Right		0.24	0.66	0.05*
Left		0.33	0.59	1.00

* Significant at the 0.05 level

#### Metabolic relationship in the cortical–subcortical–cerebellar circuitry implicated in cognitive dysmetria

In the unidirectional relationship between the cerebellum–thalamus–frontal and parietal cortices, significant differences were noted in the subcortical–cortical relationships (i.e., the thalamus–parietal relationships) [Table 3]. This difference in parietal–thalamic relationship (*P*- 0.01, 0.001) persisted after correcting for multiple comparisons.

**Table 3:** Differences in inter-regional metabolic relationships in the circuit for cognitive dysmetria (Chow test with Bonferroni corrections)

Afferent	Efferent	Regression slope coefficients		*P*-value
		Cannabis	Non-drug using controls	
Thalamus	Cerebellum			
Right		0.54	0.74	1.00
Left		0.003	0.58	1.00
Frontal cortex	Thalamus			
				
Right		0.29	0.82	0.21
Left		0.11	0.61	0.12
Parietal cortex	Thalamus			
Right		0.11	0.76	0.01*
Left		−0.005	0.60	0.001**

* Significant at the 0.01 level (2-tailed)

** significant at the 0.05 level (2-tailed)

**Figure 2:**
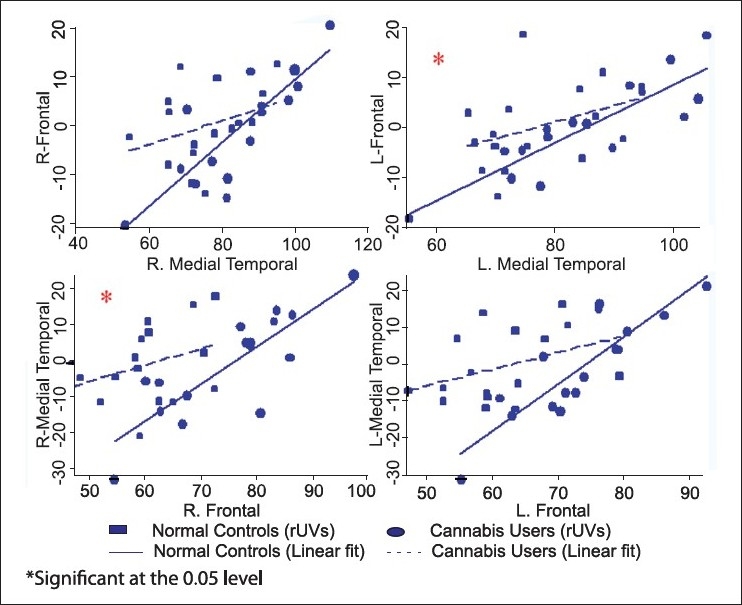
Differences in inter-regional metabolic relationships in the motive circuit for substance dependence (Chow Test with Bonferroni corrections); medial temporal - frontal in the upper layer and frontal-medial temporal in the lower layer

**Figure 3:**
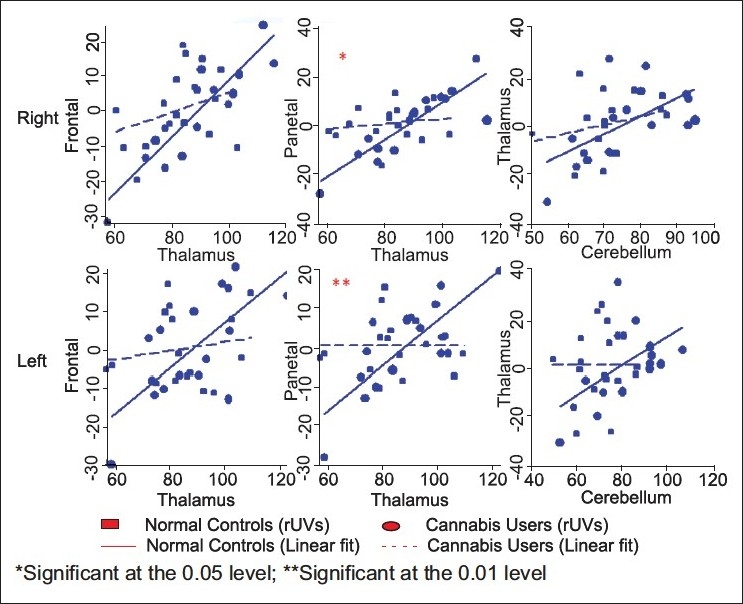
Differences in inter-regional metabolic relationships in the circuit for cognitive dysmetria (Chow Test with Bonferroni corrections) on the right (upper layer) and left (lower layer) sides

Figure 2 illustrates the afferent (upper row) and efferent (lower row) relationship of the frontal region with the medial temporal region. Each graph in upper row of figure 2 plots the rUVs of the frontal region (dependent variable) on the *Y*-axis and rUVs of the medial temporal region (independent variable) on the *X*-axis. In addition, it also includes two linear best fits based on linear regressions, one for each group. Figure 3 provides similar plots for the unidirectional relationships from the cerebellum-thalamus-frontal and parietal regions (independent variable on the *X*-axis) to other ROIs (dependent variable on the *Y*-axis). In all these, the inter-regional relationships were more negative in the cannabis users. In other words, cannabis users showed a significantly smaller correlation (as measured by the slope-coefficient of the regression) between the various ROIs than the control group.

In order to understand the influence of amount of cannabis consumption on the results that were statistically significant, we divided individuals with cannabis dependence into two groups based their CCC (high and low). The results were mixed and not statistically significant. In the substance dependence circuit, the significance was positively correlated with increasing CCC, while no trends emerged in the cognitive dysmetria circuit. These results need to be interpreted with great caution as the sample sizes in the two groups were very small.

## DISCUSSION

Lower resting global cerebral blood flow has been reported in abstinent cannabis users as compared to non-users.[12] Similar to this, in our study, we noted decreased glucose uptake in most brain regions among cannabis users. Additionally, we also noted differences in the lateral temporal uptakes, consistent with the effects of cannabis effect on temporal auditory areas.[13] However, some of our findings were contrary to earlier observations. For example, increased bilateral medial temporal uptake has been reported in cannabis intoxication[13,14] and supports the effects of cannabis on emotions and mood. In our study, glucose uptake was significantly lower in the medial temporal lobes among cannabis users as compared to non-drug using controls. Another consistent finding that was not replicated in our study is difference in cerebellar uptake.[14,15] The most likely explanation for these differences is that our study differs from the earlier mentioned studies in the temporal stage of cannabis use. The last cannabis consumption in our study was 10–12 hours prior to the scan, while in the earlier studies, patients were scanned within 30–40 minutes of cannabis consumption. This difference in time from cannabis consumption is most likely responsible for the differences in metabolism that we noticed in our study. It can be speculated that the subjects in our study were in a stage of subclinical cannabis withdrawal (subjects denied any subjective withdrawal symptoms), which is reflected in a decreased medial temporal uptake.

Cannabis intoxication has been known to result in dose-dependent changes in regional blood flow.[16] In our study, however, the uptake values were not influenced by either the duration or the amount of cannabis consumed. Further, Chang *et al*.[17] have suggested that age at first consumption may affect neural changes noted in cannabis users. We were, however, not able to replicate this observation.

Neuronal integrity is important for a number of cognitive and emotional tasks. Neural pathways in the medial temporal structures (nucleus accumbens, amygdala), frontal and ventral tegmental areas have also been identified in the mechanisms of substance dependence. A number of pathways involving the medial temporal, frontal, parietal and cerebellar structures (limbic, neocortical and cortical–subcortical–cerebellar networks)[9,17] have been implicated in various symptoms of schizophrenia. These formed the basis of our remaining hypotheses. In agreement with our second hypothesis and consistent with the diagnosis of substance dependence, cannabis users exhibited different metabolic relationships between the frontal and medial temporal regions. Surprisingly, differences were also noted in the parieto-thalamic portion of the cortical–subcortical–cerebellar circuit, suggesting that at least a part of the cortico-subcortical relationship is altered among cannabis users. This disproves our third hypothesis.

A Diffusion tensor imaging study by DeLisi *et al*.[18] concluded that moderate cannabis use does not affect neuronal integrity in growing adolescent brains. More recent work by Ashtari *et al*.[19] contradicts this and suggests that cannabis can indeed affect developing neurons particularly in the fronto-temporal connection. Chang *et al*.[20] noted that individuals with cannabis dependence recruit cognitive “reserve” regions to compensate for disrupted visual attention networks. Our findings of impaired cortical–subcortical–cerebellar circuit indicate toward a disruption in one of the cognitive networks, more specifically cognitive dysmetria. While this finding suggests that cannabis dependence can lead to cognitive dysmetria similar to schizophrenia, it does not conclusively establish that cannabis use leads to schizophrenia.

### Influence of nicotine

One factor that needs to be considered while interpreting these results is the role of nicotine. Nicotine use decreases global cerebral metabolism and increases normalized glucose metabolism in the inferior frontal cortex, posterior cingulate gyri, and the thalamus.[21] In order to examine the influence of nicotine on our results, we decided to conservatively assume that comorbid nicotine use indeed influenced the differences in metabolism between cannabis users and non-drug users and that nicotine was being abused only by individuals with cannabis dependence. Considering this, nicotine could have contributed to the global decrease in metabolism. However, the influence of nicotine on individual ROIs can be ruled out to some extent. Glucose uptake in the thalamus and medial temporal regions was low in cannabis users as compared to the normal individuals, which is contrary to the effects of nicotine. Nicotine has a half-life of 1 hour, and most work[22] on the influence of nicotine on cerebral blood flow and metabolism have assumed that the influence of nicotine wears off in approximately this time period. Participants were instructed to refrain from nicotine use in the 8–10 hours prior to the scanning process; however, nicotine dependence was not strictly controlled for. However, regional metabolism in our study could have been influenced by a craving for nicotine, although patients denied any subjective experience of craving.

### Limitations

The study was limited in that details regarding cannabis consumption were based on participant self-report.[23] Further, we have reported the quantity of cannabis in terms of amount of money spent rather than the weight, as the amount spent reflects the true potency. The most accurate assessment would have been plasma Tetrahydrocannabinol levels, which we were unable to perform. This study is also technically limited by the application of the manual method of analysis instead of Statistical Parametric Mapping (SPM), which also constrains anatomical micro-definitions. The main reason for this was that our images were not in the DICOM format essential for using SPM. However, in order to avoid the operator-based errors of this method, we ensured that the same blinded neurodiagnostician reported all the scans. Additionally, some studies[20,24] have suggested that the neurotoxic effects of cannabis may be reversible. The present study is a cross-sectional one; hence, definite conclusions regarding causality cannot be drawn. Longitudinal studies examining these relationships are required to clarify if the alterations in neuronal relationships are the result of cannabis use.

To conclude, the study reaffirms the global decrease in glucose metabolism associated with cannabis consumption. Importantly, this study confirms that cannabis dependence can alter metabolic relationships between important cerebral regions, indicating changes in neuronal circuits. If the disruption in relationships between the various regions noted in cannabis users in our study persisted as long-term changes, this could explain not only cannabis dependence but also the cognitive and emotional disorders arising from cannabis use.
